# Changing the clinical course of glioma patients by preoperative motor mapping with navigated transcranial magnetic brain stimulation

**DOI:** 10.1186/s12885-015-1258-1

**Published:** 2015-04-08

**Authors:** Sandro M Krieg, Nico Sollmann, Thomas Obermueller, Jamil Sabih, Lucia Bulubas, Chiara Negwer, Tobias Moser, Doris Droese, Tobias Boeckh-Behrens, Florian Ringel, Bernhard Meyer

**Affiliations:** 1 Department of Neurosurgery, Klinikum rechts der Isar, Technische Universität München, Ismaninger Str. 22, 81675 Munich, Germany; 2 TUM-Neuroimaging Center, Klinikum rechts der Isar, Technische Universität München, Ismaninger Str. 22, 81675 Munich, Germany; 3 Section of Neuroradiology, Department of Radiology, Klinikum rechts der Isar, Technische Universität München, Ismaninger Str. 22, 81675 Munich, Germany; 4 Department of Anesthesiology, Klinikum rechts der Isar, Technische Universität München, Ismaninger Str. 22, München, 81675 Germany

**Keywords:** Brain tumor, Matched pair, Preoperative mapping, Rolandic region, Transcranial magnetic stimulation

## Abstract

**Background:**

Mapping of the motor cortex by navigated transcranial magnetic stimulation (nTMS) can be used for preoperative planning in brain tumor patients. Just recently, it has been proven to actually change outcomes by increasing the rate of gross total resection (GTR) and by reducing the surgery-related rate of paresis significantly in cohorts of patients suffering from different entities of intracranial lesions. Yet, we also need data that shows whether these changes also lead to a changed clinical course, and can also be achieved specifically in high-grade glioma (HGG) patients.

**Methods:**

We prospectively enrolled 70 patients with supratentorial motor eloquently located HGG undergoing preoperative nTMS (2010–2014) and matched these patients with 70 HGG patients who did not undergo preoperative nTMS (2007–2010).

**Results:**

On average, the overall size of the craniotomy was significantly smaller for nTMS patients when compared to the non-nTMS group (nTMS: 25.3 ± 9.7 cm^2^; non-nTMS: 30.8 ± 13.2 cm^2^; p = 0.0058). Furthermore, residual tumor tissue (nTMS: 34.3%; non-nTMS: 54.3%; p = 0.0172) and unexpected tumor residuals (nTMS: 15.7%; non-nTMS: 32.9%; p = 0.0180) were less frequent in nTMS patients. Regarding the further clinical course, median inpatient stay was 12 days for the nTMS and 14 days for the non-nTMS group (nTMS: CI 10.5 – 13.5 days; non-nTMS: CI 11.6 – 16.4 days; p = 0.0446). 60.0% of patients of the nTMS group and 54.3% of patients of the non-nTMS group were eligible for postoperative chemotherapy (OR 1.2630, CI 0.6458 – 2.4710, p = 0.4945), while 67.1% of nTMS patients and 48.6% of non-nTMS patients received radiotherapy (OR 2.1640, CI 1.0910 – 4.2910, p = 0.0261). Moreover, 3, 6, and 9 months survival was significantly better in the nTMS group (p = 0.0298, p = 0.0015, and p = 0.0167).

**Conclusions:**

With the limitations of this study in mind, our data show that HGG patients might benefit from preoperative nTMS mapping.

## Background

Many studies have now shown that surgical neuro-oncology requires an optimal extent of resection (EOR) since it directly correlates with survival of glioma patients. Thus, gross total resection (GTR) has to be the surgical aim for neurosurgeons when treating glioma patients [1-3]. Especially when affecting or neighboring the motor cortex, GTR is still a neurosurgical quest requiring a multimodal approach of preoperative mapping and intraoperative mapping and monitoring. Intraoperatively, we already have well-established techniques to monitor functional integrity of the motor strip and corticospinal tract (CST) such as continuous motor evoked potential (MEP) monitoring as well as cortical (DCS) and subcortical electrical stimulation [4-6]. Besides functional magnetic resonance imaging (fMRI) and magnetoencephalography (MEG) we now have another modality at hand for preoperative mapping: navigated transcranial magnetic brain stimulation (nTMS).

In general, transcranial magnetic stimulation (TMS) penetrates the skull and induces an electric field within the motor cortex, which then causes neuronal depolarization and therefore an action potential that can be measured as a MEP [7]. By combining the TMS technique with a neuronavigation unit, we are now able to navigate the TMS coil and thus its site of cortical stimulation [8]. In this context, nTMS was repeatedly shown to correlate well with intraoperative DCS and already demonstrated to be superior to fMRI and MEG [9-11]. But more importantly, nTMS has been proven to not only influence surgical indication and planning but also lead to an increased rate of GTR and to a reduced rate of surgery-related paresis [12,13]. However, this was shown in a comparatively inhomogeneous cohort suffering from different kinds of brain lesions and without taking into account the further clinical course [12,13]. Thus, further investigation in a more homogeneous patient cohort including analysis of the longer clinical course seems reasonable.

This study was therefore designed to compare the clinical course of patients with motor eloquently located supratentorial high-grade gliomas (HGG) who underwent preoperative nTMS with a historic control group of patients who were operated on without nTMS data by a matched pair analysis.

## Methods

### Patients

Indication for nTMS and intraoperative neuromonitoring (IOM) due to topographic association between tumor and precentral gyrus was assessed by magnetic resonance imaging (MRI) for all patients. Seventy consecutive patients suffering from motor eloquently located supratentorial HGG (16 WHO grade III and 54 WHO grade IV gliomas) were enrolled between 2010 and 2014, received preoperative nTMS motor mapping, and underwent craniotomy in our department. In order to create a control group, this prospectively enrolled consecutive cohort was matched with HGG patients (14 WHO grade III and 56 WHO grade IV gliomas) operated on from 2007 to 2010 in our department by the same group of surgeons. A minority of the enrolled patients was also included in a recent trial of our group [13].

Matching criteria were tumor location, preoperative paresis, and histology. Group characteristics and statistical data of both groups are provided in Table 1. Each step of data analysis was performed by investigators blinded to the assigned group for each patient (NS, TO).

**Table 1:** **Patient data**

		nTMS	non-nTMS	p-value
Mean age (years)		58.0 ± 13.8	60.3 ± 14.7	0.3328
Gender (%)	Male	64.3	64.3	1.0000
	Female	35.7	35.7	
Median preoperative Karnofsky performance status (%)		80.0 (95% CI 76.7 – 83.3)	80.0 (95% CI 76.7 – 83.3)	0.3351
Preoperative paresis (%)	None	68.6	71.4	0.5079
	Mild	24.3	25.7	
	Severe	7.1	2.9	
Histology (%)	WHO grade III	22.9	20.0	0.6804
	WHO grade IV	77.1	80.0	
Mean tumor diameter (cm)		4.1 ± 2.7	4.2 ± 1.8	0.9328
Mean follow-up (months)		12.8 ± 10.4	17.6 ± 19.5	0.0742

Detailed overview on age, gender, Karnofsky performance status (KPS), preoperative neurological status, histology, mean tumor diameter on axial slices, and mean follow-up of the nTMS compared to the non-nTMS group. Preoperative paresis: none = no paresis, mild = BMRC grade of muscle strength ≥ 4-/5, severe = BMRC grade of muscle strength ≤ 3/5.

### Ethical standard

The presented study is in accordance with ethical standards outlined in the Declaration of Helsinki. The study protocol was also approved by the local institutional review board of the TU München (registration number: 2793/10). Every patient gave written informed consent prior to the nTMS examination.

### Clinical and oncological assessment

All clinical assessment was done blinded to the nTMS data. Each patient underwent a detailed examination according to a standardized protocol including sensory function, coordination, muscle strength, and cranial nerve function. Muscle strength was assessed according to the British Medical Research Council (BMRC) scale. The protocol was established in 2006 as clinical routine in our department. Postoperatively, the neurological status was again assessed for each patient directly after anesthesia and daily from the first postoperative day until discharge, again at 6–8 weeks postoperatively, and during follow-ups every 3–12 months depending on WHO grade. We clearly differentiated between permanent and temporary paresis due to surgery. Any new or aggravated paresis due to surgery that did not resolve to the preoperative status during the regular 8-week follow-up interval was defined as a new permanent paresis. A temporary paresis, however, was defined as any new or worse surgery-related paresis, which resolved at least during the 8-week follow-up interval. Nonetheless, as a standard of care, we perform a direct postoperative computed tomography (CT) scan or even MRI in all glioma patients who present with a new paresis immediately after anesthesia.

We moreover analyzed the postoperative course of all patients including Karnofsky performance status scale (KPS) as well as eligibility for postoperative chemotherapy and radiotherapy. In addition, the rate of postoperative infection in terms of meningitis, which was diagnosed by lumbar puncture in case of justified clinical suspicion, was evaluated.

### Magnetic resonance imaging

All MRI scans were performed before and after surgery in all patients with a 3 Tesla MR scanner with an 8-channel phased array head coil (Achieva 3 T, Philips Medical Systems, The Netherlands B.V.). Our standard included contrast-enhanced 3D gradient echo sequence, FLAIR, and diffusion tensor imaging (DTI). The contrast-enhanced 3D gradient echo sequence dataset was transferred to the nTMS system (eXimia 3.2 and eXimia 4.3, Nexstim Oy, Helsinki, Finland). The day after surgery all patients underwent another MRI scan to evaluate the EOR. The protocol included T1 sequences with and without contrast, FLAIR, and diffusion-weighted imaging (DWI) to search for postoperative ischemic events. MRI scans were also performed during regular follow-up every 3–12 months depending on WHO grade and current oncological treatment. Since recurrent gliomas might affect the neurological status, all follow-up MRI scans were cautiously reviewed for recurrent tumors since the neurological status was only considered during progression-free survival.

The evaluation of all MRI data was performed by at least two board certified neuroradiologists. Regarding the EOR, the results of this evaluation were discussed in an imaging meeting of board certified neuroradiologists and neurosurgeons, and a final decision was made based on the scanning sequences described. GTR according to MRI was assessed when there was no residual tumor tissue identified on postoperative scans after careful comparison to preoperative imaging. Furthermore, the rate of complications (increasing edema, ischemia, bleeding, cerebrospinal fluid circulation = CSF dysfunction) according to MRI was evaluated for later comparison between the nTMS and non-nTMS group.

### Navigated transcranial magnetic stimulation

In this study, a nTMS system (eXimia 3.2 and eXimia 4.3, Nexstim Oy, Helsinki, Finland) consisting of a biphasic figure-8 TMS coil with a 50 mm radius as stimulator combined with an infrared tracking unit (Polaris Spectra, Waterloo, Ontario, Canada) was used as outlined in earlier reports [10,11,14]. By using a 3D gradient echo sequence we can visualize the stimulated cortical spots and therefore investigate the distribution of motor function within the human brain.

All enrolled HGG patients of the nTMS group underwent mapping of the primary motor cortex by a standardized protocol by an experienced investigator using 110% resting motor threshold (rMT) for the upper extremity and 130% rMT for the lower extremity in 3 to 5 mm steps perpendicular to the sulci until stimulation did not elicit any further MEP in any direction as also published by many groups [9,11,13-16]. Each cortical spot at which a MEP was evoked was regarded as a part of the motor cortex of the mapped muscles and exported from the nTMS system via DICOM standard to the intraoperative neuronavigation system (BrainLAB AG, Feldkirchen, Germany).

### Surgical setup

Surgical technique did not vary between groups. The resection of all 140 HGG was supported by monopolar DCS in order to monitor the motor system by MEPs as described in earlier reports [12,17,18].

As a second intraoperative modality, neuronavigation was used throughout (Vector Vision 2®, Vector Vision Sky®, and Curve; BrainLAB AG, Feldkirchen, Germany) in all patients. In the nTMS group, the positive nTMS points were visualized as 3D objects by simple auto segmentation within the neuronavigational data set (BrainLAB iPlan® Net Cranial 3.0.1; BrainLAB AG, Feldkirchen, Germany). Positron emission tomography (PET) was fused and integrated into the data set as well. The inclusion of nTMS data as 3D objects in the neuronavigational planning required about 2 to 5 minutes for each case.

Additional techniques, such as intraoperative MRI or ultrasonography, were not used during surgery, and five-aminolevulinic acid (5-ALA) was only used infrequently dependent on the surgeon`s preoperative decision. However, there was no difference in the usage frequency of 5-ALA between the nTMS and the non-nTMS group.

### Statistical analysis

Chi-square or Fisher Exact test were performed to test the distribution of several attributes. The Mann–Whitney-Wilcoxon test for multiple comparisons on ranks for independent samples (non-parametric distribution) and the t-test (for parametric distribution) were used for testing of differences between 2 groups.

All results are presented as mean ± standard deviation (SD) and as odds ratios (OR) with 95% confidence intervals (CI) (GraphPad Prism 5.0c, La Jolla, CA, USA). The level of significance was 0.05 for each statistical test (two-sided).

## Results

### Preoperative nTMS mapping

All 70 consecutive HGG patients of the nTMS group underwent preoperative mapping of the primary motor cortex. Mean rMT of this cohort was 33.3 ± 8.2% maximum stimulator output. Regarding potential nTMS-related discomfort, no patient described the stimulation as painful or asked for reduction of stimulation intensity due to pain. In addition, no adverse events, especially seizures, were observed.

### Influence on surgery

#### Duration of surgery

Duration of surgery was 201.0 ± 57.0 min (median 198.5 min, range 81.0 – 380.0 min) for nTMS and 208.9 ± 65.5 min (median 192.0 min, range 101.0 – 401.0 min) for non-nTMS patients (p = 0.4495).

#### Craniotomy size

**Figure 1:**
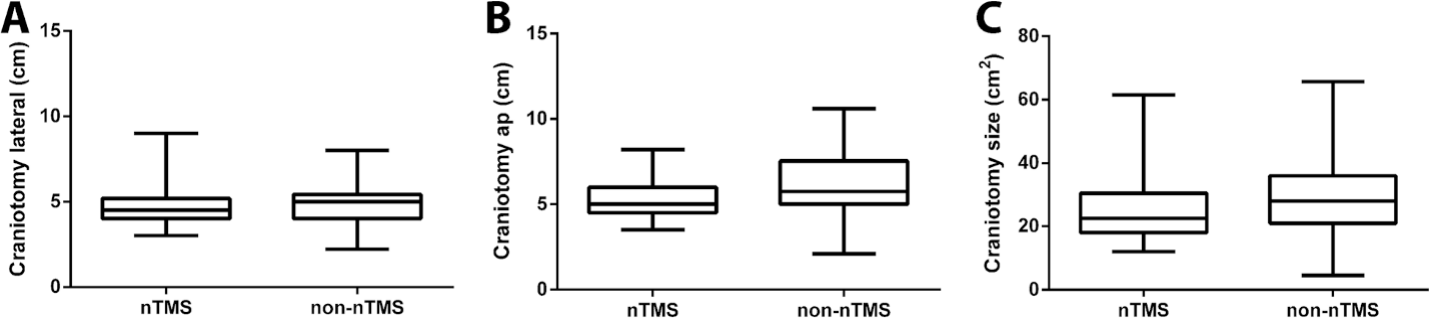
**Size of craniotomy.** Boxplot of craniotomy extension for the nTMS compared to the non-nTMS group with median, min-, and max-whiskers, and quartile-boxes for the lateral direction (**A**; p = 0.2924), anterior-posterior direction (**B**; p = 0.0014) and overall size of the craniotomy (**C**; p = 0.0058).

The lateral extension of the bone flap was 4.8 ± 1.1 cm (median 4.5 cm, range 3.0 – 9.0 cm) for nTMS and 5.0 ± 1.1 cm (median 5.0 cm, range 2.2 – 8.0 cm) for non-nTMS patients (p = 0.2924; Figure 1A). Anterior-posterior (AP) extent of the craniotomy was 5.2 ± 1.1 cm (median 5.0 cm, range 3.5 – 8.2 cm) for nTMS and 6.1 ± 1.9 cm (median 5.8 cm, range 2.1 –10.6 cm) for non-nTMS patients (p = 0.0014; Figure 1B). Resulting overall size of the craniotomy was 25.3 ± 9.7 cm^2^ (median 22.5 cm^2^, range 12.0 – 61.6 cm^2^) for nTMS and 30.8 ± 13.2 cm^2^ (median 28.0 cm^2^, range 4.6 – 65.7 cm^2^) for non-nTMS patients (p = 0.0058; Figure 1C). According to p-values, there was a significant difference in both the AP extent of the craniotomy as well as the overall craniotomy size between both groups.

### Karnofsky performance status scale

Median pre- and postoperative KPS were highly comparable in both groups without showing statistically significant differences (Tables 1 and 2).

**Table 2:** **Postoperative course**

		nTMS	non-nTMS	p-value
Median postoperative Karnofsky performance status (%)		80.0 (95% CI 76.1 – 83.9)	80.0 (95% CI 76.1 – 83.9)	0.3311
Median inpatient stay (days)		12.0 (95% CI 10.5 – 13.5)	14.0 (95% CI 11.6 – 16.4)	0.0446
Residual tumor (%)		34.3	54.3	0.0172
Unexpected residual (%)		15.7	32.9	0.0180
Surgery-related paresis (%)	None	78.6	70.0	0.1138
	Transient	8.6	4.3	
	Permanent	12.9	25.7	
Surgery-related complications on MRI (%)	Increasing edema	6.7	5.4	0.5154
	Ischemia	18.7	9.5	
	Bleeding	13.3	17.6	
	CSF circulation dysfunction	1.3	2.7	

This table provides information about the postoperative course of the nTMS compared to the non-nTMS group, including Karnofsky performance status (KPS), inpatient stay, residual tumor, unexpected residual, surgery-related paresis, and surgery-related complications as shown by MRI.

### Motor status

#### Preoperative paresis

Overall, preoperative motor deficits were found at comparable frequency within both patient groups since being part of the matching algorithm. Table 1 provides detailed information about the distribution and degree of preoperative motor impairment.

#### Overall motor outcome

Mild postoperative paresis was found in 13 patients of the nTMS group (18.6%), whereas 18 subjects of that group (25.7%) showed a severe degree of motor function impairment. Concerning the non-nTMS group, 15 subjects (21.4%) were suffering from mild paresis, and 18 patients (25.7%) were diagnosed with severe paresis postoperatively. However, there was no significant difference between the nTMS group and non-nTMS group (p = 0.9069).

When comparing the degree of pre- and postoperative paresis, 1 patient of the nTMS group (1.4%) improved, whereas 15 subjects of that group (21.4%) got worse. Regarding subjects that were not mapped by nTMS preoperatively, 2 patients (2.9%) improved, whereas 21 patients (30.0%) were suffering from increased motor impairment. Again, differences between both groups were not significant (p = 0.4028).

#### Motor status during follow-up

During follow-up, 14 subjects of the nTMS group (20.0%) presented with mild and 14 subjects (20.0%) presented with severe paresis. In contrast, a number of 11 patients of the non-nTMS cohort (15.7%) were suffering from mild paresis, whereas 19 subjects (27.1%) showed a severe degree of motor impairment. Regarding these results, statistical analysis did not show significance (p = 0.5581).

When comparing immediate postoperative motor outcome with motor status during follow-up, 8 patients of the nTMS group (11.4%) improved, while motor function of 2 nTMS patients got worse (2.9%). In the group of non-nTMS patients, 5 subjects (7.1%) increased in motor function, while 4 patients (5.7%) were suffering from increasing paresis. Again, the difference between groups failed to be significant (p = 0.5048).

**Figure 2:**
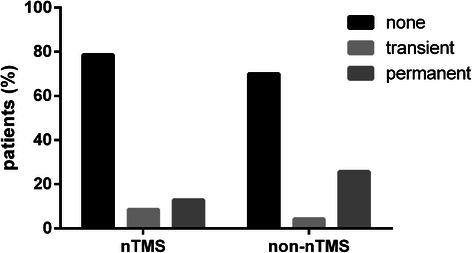
**Surgery-related paresis on long-term follow-up.** The graph illustrates the percentage of patients suffering from a transient paresis to the percentage of patients who were diagnosed with a new permanent paresis on long-term follow-up for the nTMS group in comparison to the non-nTMS group (p = 0.1113).

Furthermore, permanent surgery-related paresis was found more frequently in subjects of the non-nTMS cohort, while transient motor deficits occurred more often in nTMS patients (Table 2, Figure 2). Although the results show a clear trend, the difference in surgery-related paresis between groups eventually did not reach statistical significance (Table 2).

**Figure 3:**
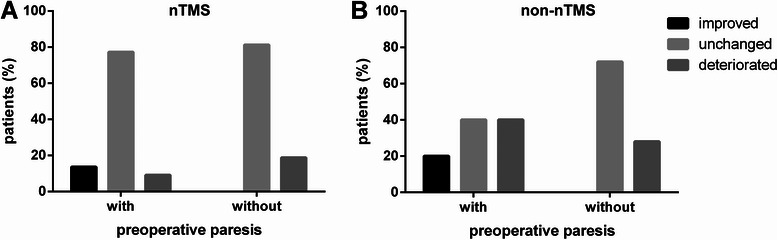
**Permanent surgery-related deficit depending on preoperative paresis.** The bar chart compares the percentage of patients with and without a preoperative paresis in both the nTMS (**A**; p = 0.0239) and the non-nTMS group (**B**; p = 0.0015), which can be improved, unchanged, or deteriorated on long-term follow-up compared to the preoperative state.

With regard to the clinical course between preoperative status and follow-up, 3 nTMS patients (4.3%) improved in motor function, while 11 subjects (15.7%) deteriorated. Within the group of non-nTMS patients, motor function increased in 4 subjects (5.7%) and decreased in 22 subjects (31.4%; Figure 3).

### Peri- and postoperative complications on MRI

There was no significant difference in the distribution of peri- and postoperative complications between both groups (Table 2).

### Postoperative infection

Within the nTMS group, postoperative infection was observed in 6 patients (8.6%), whereas it occurred in 9 subjects of the non-nTMS cohort (12.9%, p = 0.4124).

### Extent of resection and persisting surgery-related deficit

Both residual tumor tissue and unexpected residual tumor were found significantly more frequently on postoperative MRI within the non-nTMS group compared to nTMS patients (Tables 2 and 3).

### Inpatient stay

**Figure 4:**
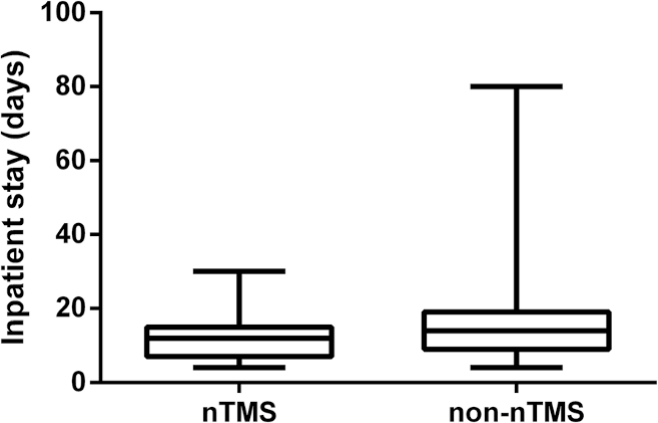
**Inpatient stay.** Boxplot illustrating the duration of inpatient stay for the nTMS group in comparison to the non-nTMS group (p = 0.0446).

In total, patients of the nTMS group showed a significantly shorter inpatient stay than patients of the non-nTMS cohort (Table 2; Figure 4).

### Adjuvant therapy

#### Chemotherapy

Overall, there was no statistically significant difference in the application of postoperative chemotherapy in both groups (Table 4).

Within the nTMS group, 42 patients (60.0%) were treated by adjuvant chemotherapy, which consisted of temozolomide (36 cases, 51.4% of patients), temozolomide + bevacizumab (4 cases, 5.7%), temozolomide + CCNU (1 case, 1.4%), or bevacizumab + CCNU + procarbazine (1 case, 1.4%). Concerning patients of the non-nTMS group, 38 (54.3%) received adjuvant chemotherapy. Therefore, temozolomide (30 cases, 42.9% of patients), temozolomide + bevacizumab (5 cases, 7.1%), temozolomide + CCNU (1 case, 1.4%), temozolomide + capecitabin (1 case, 1.4%), or temozolomide + ACNU (1 case, 1.4%) were applied.

With regard to the length of adjuvant chemotherapy, patients of the nTMS group were treated 2.5 ± 2.5 months (median 2.0 months, range 0.0 – 7.0 months), whereas subjects of the non-nTMS group received treatment for 2.2 ± 2.6 months (median 1.0 month, range 0.0 – 7.0 months; p = 0.5012).

#### Radiotherapy

Significantly more patients underwent postoperative radiotherapy in the nTMS group compared to the non-nTMS cohort (Table 4).

Yet, there was no significant difference in the applied radiation dose (nTMS: 53.6 ± 10.4 gray, median 60.0 gray, range 30.0 – 60.0 gray; non-nTMS: 57.9 ± 8.6 gray, median 60.0 gray, range 30.0 – 70.0 gray; p = 0.1821).

### Time to follow-up

Table 1 provides information about mean times to follow-up for both patient groups. According to these data, differences between groups were not significant.

### Survival rates

**Figure 5:**
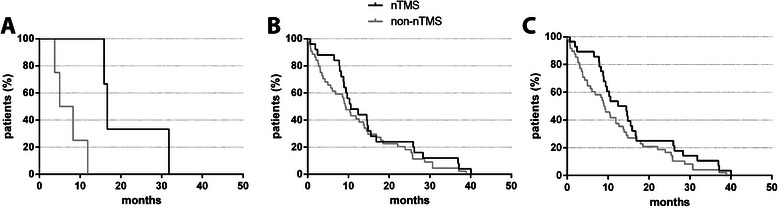
**Survival.** Overall survival shown via Kaplan Meier curve for both groups in WHO grade III (**A**; p = 0.0322), WHO grade IV (**B**; p = 0.3196), and all patients (**C**; p = 0.1310).

In general, mean overall survival was better in the nTMS group, but there was no significant difference between both groups (Table 5, Figure 5). The corresponding median overall survival was 13.5 months for the nTMS and 9.1 months for the non-nTMS group.

When only taking into account mean overall survival data of WHO grade III tumor patients, subjects of the nTMS group survived longer than patients of the non-nTMS cohort, and this difference was statistically significant (Table 5). Furthermore, median overall survival was 16.7 months in the nTMS and 6.6 months in the non-nTMS group. Yet, due to the limited number of deaths in the WHO grade III tumor patients, these results have to be regarded with very limited impact.

With regard to WHO grade IV tumor patients, nTMS subjects’ mean overall survival was longer than those of the patients of the non-nTMS group but without reaching statistical significance (Table 5). In this context, the median overall survival was 10.6 months for the nTMS and 9.3 months for the non-nTMS cohort. Furthermore, WHO grade IV tumor patients of the nTMS group showed a significantly higher survival rate after 3, 6, and 9 months (Table 5).

**Table 3:** **Extent of resection and surgery-related new permanent paresis**

	GTR		STR	
	nTMS	non-nTMS	nTMS	non-nTMS
New permanent paresis (%)	17.4	28.1	12.5	34.2
No new permanent paresis (%)	82.6	71.9	87.5	65.8
p-value	0.2587		0.0570	

The percentage of patients with and without new permanent paresis after gross total resection (GTR) or subtotal resection (STR) according to postoperative MRI for both the nTMS and non-nTMS groups.

## Discussion

In general, both groups were highly comparable in tumor entity, size, patient age, KPS, and preoperative motor deficit (Table 1). Yet mean follow-up was different in both groups due to the earlier date of surgery in the non-nTMS group with a considerable number of survivors among the WHO grade III patients (Table 1).

### Craniotomy size

According to the results of this study, there was a statistically significant difference in the AP extent of the craniotomy and the overall craniotomy size between both patient cohorts (Figure 1). Therefore, it seems to be likely that nTMS for preoperative motor mapping is able to minimize the required size of craniotomy, probably due to the absent necessity to perform extensive intraoperative mapping. The surgeon’s task then is just to confirm nTMS data by circumscribed DCS mapping, which allows craniotomy sizes to be smaller especially in the AP direction, which is usually larger to reach the rolandic region for intraoperative DCS mapping [10]. This finding is in accordance with a recently published study, which showed that nTMS motor mapping can decrease the size of craniotomy in a group of patients suffering from different brain tumor entities [13]. However, we are not aware of publications showing that smaller craniotomies are directly linked to better patient outcomes or increased safety. Thus, future studies are needed to assess whether smaller craniotomies due to preoperative nTMS motor mapping can influence such parameters, too.

### Residual tumor

Overall, patients of the nTMS cohort were diagnosed with a lower rate of residual tumor tissue according to postoperative MRI scans in comparison to non-nTMS subjects, which means that GTR was more often achieved in nTMS patients (Table 2). This difference has also been observed in two recently published studies investigating more inhomogeneous cohorts of brain tumor patients [12,13]. Furthermore, unexpected residual was significantly more frequently observed within the non-nTMS patients than in their nTMS counterparts (Table 2).

Comparing these findings with current literature, Duffau et al. [5], but also a recent meta-analysis by De Witt Hamer et al. [19] reported an increased EOR by the use of intraoperative mapping for low-grade [5] and infiltrative [19] gliomas respectively, which proves the value of functional mapping per se no matter whether it is performed pre- or intraoperatively [5,19]. The relationship between preoperative nTMS motor mapping and lower residual tumor rates suggests that the intraoperative visualization of nTMS mapping results on the neuronavigation system is likely to increase the surgeons’ confidence in the neuroanatomy, as repeatedly reported, and therefore leads to a more radical resection [10]. This coherence was also repeatedly observed in IOM investigations [5,10,20].

### Surgery-related paresis

Concerning surgery-related paresis, Tables 2 and 3 show that surgery-related permanent paresis was less frequent in the nTMS group, especially for subtotal resection (STR). However, it is important to state that this difference between the nTMS and non-nTMS group failed to reach statistical significance (Tables 2 and 3). Nevertheless, a similar finding was also shown in two recently published reports including patients suffering from different entities of intracranial lesions, which both indicated that the rates of new postoperative motor deficits are lower in patients undergoing nTMS motor mapping prior to surgery [12,13].

### Clinical course

#### Karnofsky performance status scale

Regarding the KPS, pre- and postoperative scores were highly comparable in both groups (Tables 1 and 2), which means that preoperative nTMS motor mapping did not have a significant impact on postoperative KPS. Frey et al. [12] also showed that nTMS is not likely to change the average KPS in a significant dimension for patients with various intraparenchymal lesions [12].

It is already known that KPS can serve as a prognostic indicator for survival in glioma patients [21-25]. Therefore, a positive effect of nTMS for preoperative motor mapping on KPS would be of distinct clinical impact. However, as indicated by the less frequent surgery-related permanent paresis (Tables 2 and 3), nTMS still changes the postoperative clinical course of HGG patients in a positive way, but this does not seem to affect KPS scores significantly in comparison to the non-nTMS cohort, probably due to the relatively broad categorization of the KPS, which primarily covers obvious clinical changes.

#### Inpatient stay

Patients of the non-nTMS group showed a significantly longer inpatient stay than patients of the nTMS cohort (Table 2, Figure 4). Since standard of care did not change since 2006 in our department, this observation could be attributed to a lower rate of surgery-related paresis, which qualifies more patients for further treatment on an outpatient basis. However, this interpretation has to be confirmed by future multicenter studies including more patients.

#### Adjuvant chemo- and radiotherapy

Overall, a higher rate of nTMS patients underwent adjuvant radiotherapy treatment in comparison to subjects of the non-nTMS group (Table 4). Since therapeutic protocols did not change in the observed period, it could be likely that this is the result of a lower surgery-related deficit rate among nTMS patients (Table 2). Again, further investigations including more patients are needed to support this explanation.

**Table 4:** **Additional therapy**

		nTMS	non-nTMS	p-value
Chemotherapy (%)	WHO grade III	75.0	64.3	0.4945
	WHO grade IV	55.6	51.8	
Radiotherapy (%)	WHO grade III	62.5	28.6	0.0261
	WHO grade IV	68.5	53.6	

This table gives information about the percentage of patients of the nTMS and non-nTMS group that received adjuvant chemo- and/or radiotherapy in relation to the WHO grade of the tumor respectively.

#### Survival

Even with the comparatively small sample size of our report, 3, 6, and 9 month survival rates were significantly better in the nTMS cohort (Table 5). This finding seems obvious since it is the combined result of higher adjuvant therapy rates and a higher rate of GTR in the nTMS group (Tables 2 and 4). However, we encourage to carefully discussing this observation in the light of upcoming studies dealing with this issue.

**Table 5:** **Survival**

		nTMS	non-nTMS	p-value
All tumors	Overall survival (months)	15.7 ± 10.9	11.9 ± 10.3	0.1310
	3 months survival rate (%)	93.7	80.9	0.0298
	6 months survival rate (%)	88.5	62.7	0.0015
	9 months survival rate (%)	72.9	50.7	0.0167
	12 months survival rate (%)	58.7	40.3	0.0544
WHO grade III	Overall survival (months)	21.5 ± 9.0	7.2 ± 3.6	0.0322
	3 months survival rate (%)	100.0	100.0	1.000
	6 months survival rate (%)	100.0	84.6	0.1410
	9 months survival rate (%)	91.7	76.9	0.3151
	12 months survival rate (%)	80.0	69.2	0.5598
WHO grade IV	Overall survival (months)	15.1 ± 11.1	12.4 ± 10.6	0.3196
	3 months survival rate (%)	91.8	76.4	0.0332
	6 months survival rate (%)	84.6	57.4	0.0052
	9 months survival rate (%)	66.7	44.4	0.0384
	12 months survival rate (%)	52.8	33.3	0.0663

Detailed survival data including mean overall survival, 3, 6, 9, and 12 months survival rates for all tumor patients and separately for WHO grade III and IV tumor patients of both groups.

### Further non-invasive mapping modalities

There have never been so many different mapping modalities at hand as there are today. Besides nTMS, fMRI is a frequently used and broadly available technique for non-invasive cortical motor mapping. Despite the fact that resting-state as well as task-related fMRI gains increasing neuroscientific importance especially for functional connectivity analysis, the exact correlation between the fMRI signal and its neurophysiological background is still not fully understood. In that sense, fMRI only provides an indirect measure (blood oxygenation level dependent = BOLD-signal) of neurological activation reflected by increased local brain metabolism but does not measure electrophysiological function itself. However, brain metabolism regularly changes due to tumor infiltration, for instance [26,27]. As a consequence, fMRI – but also PET for the same reasons – probably lacks sufficient sensitivity and specificity to identify eloquent brain function in the vicinity of intracerebral lesions and therefore, this technique should be avoided for presurgical planning [28-31].

Another regularly used tool for motor mapping is MEG, which, according to previous comparison studies, correlates well with nTMS since its principle is also based on neurophysiology [9,32]. But, mainly due to the high costs, its distribution and availability are very limited despite its valuable characteristics as a non-invasive and reliable mapping technique [9,33].

### Limitations

#### Limitations of nTMS

Although the present study provides valuable data concerning a variety of outcome factors, we have to be aware of certain limitations of the nTMS technique itself.

The results of preoperative motor mapping by nTMS can be confounded by different factors, such as registration and navigation errors or imprecise determination of the individual rMT [13,14,34,35]. According to our experience, intraoperative brain shift does not have to be regarded as a confounder in general when fusing nTMS data with neuronavigation, because nTMS data is used to get an initial impression of the correlation between function and anatomy especially directly after opening the dura. Furthermore, the implementation of nTMS data into neuronavigation presents its second main value by identifying the precentral gyrus immediately after durotomy, which can then be identified visually for the remaining time of surgery.

#### Limitations of this particular study

The lack of randomization has to be regarded as the major limitation of the present study. In this context, patients of the non-nTMS group underwent surgery between 2007 and 2010, whereas the other cohort was motor-mapped and operated on between 2010 and 2014. Yet, the used techniques (IOM, neuronavigation) as well as the surgical team did not change significantly from 2006 to 2014 [12,13,36].

Due to the fact that patients treated within 2013 and 2014 were also incorporated into the nTMS cohort, mean follow-up of these subjects is comparatively low. Therefore, the definite benefit of nTMS within these patients has probably not yet taken effect, which can decrease the strength of results gained among nTMS patients.

Additionally, the control group of the present study was not mapped for cortical motor areas by any other neuroimaging modality like fMRI or MEG. The practical value of these techniques for functional mapping in brain tumor patients due to changed anatomy and tissue metabolism can be discussed controversially, but they represent the more established modalities used by many centers these days when compared to nTMS. For that reason, a systematic matching of the nTMS cohort to a patient group who underwent fMRI or MEG seems reasonable. However, since the aim of the current study was to distinctly focus on preoperative nTMS motor mapping and its impact on the clinical course of HGG patients, we decided to match the nTMS group with a purely non-nTMS patient cohort.

### Future impact of nTMS motor mapping on neurosurgery

The non-invasiveness and therefore presurgical applicability of nTMS is one of its main advantages: preoperative nTMS-based identification of motor areas was repeatedly reported to be helpful in surgical planning for motor eloquent lesions, because it enables the surgeon to precisely identify the cortical representations of individual muscles as many surgeons are already used to during surgery [10,14,20,37]. Additionally, thanks to nTMS mapping, we are able to assess the risk for surgery-related paresis more precisely: nTMS motor mapping allows for preoperative evaluation of each patient’s individual risk of potential surgery-related paresis, because mapping results provide precise information about the distance between the intended tumor resection border and the rolandic region for every single patient on a neurophysiological basis. More importantly, preoperative nTMS motor mapping might further improve the outcome of brain tumor patients, especially in terms of surgery-related paresis [12,13].

### Summary and significance

At least to our knowledge, this is the first study that systematically investigated the impact of preoperative nTMS-based motor mapping on different clinical outcome parameters within a homogeneous cohort of HGG patients. In this context, we were able to demonstrate that craniotomies were significantly smaller in nTMS patients, and residual tumor tissue as well as unexpected residuals were less frequent when compared to a non-nTMS control group. Regarding motor function, nTMS patients suffered less frequently from surgery-related paresis than their non-nTMS counterparts, although this difference was not statistically significant. These findings are generally in good accordance with the two recently published and aforementioned studies [12,13]. Consequently, the present study revealed that the promising results of these two publications can also be confirmed for HGG patients. Furthermore, the present study evaluated the further clinical course of the enrolled patients: median inpatient stay was shorter and radiotherapy was also possible in a higher number of patients in the nTMS group. Besides a trend towards higher mean overall survival rate in the nTMS group, there were statistically significant differences for the 3, 6, and 9 months survival in favor of the nTMS group.

Although these results are encouraging and have not been described in the context of preoperative nTMS motor mapping yet, we are distinctly aware of the limitations of the present study, which do not allow the attribution of these findings to nTMS without any doubt of possible confounders. Therefore, future studies including larger patient cohorts are highly needed to explore whether preoperative nTMS can be considered as the distinct cause for these results.

Nonetheless, this work further increases the level of evidence for preoperative nTMS-based motor mapping for rolandic brain tumor patients in a group comparison study.

## Conclusions

With the limitations of this study in mind, our data shows that HGG patients might benefit from preoperative nTMS mapping with regard to various clinical outcome parameters. Yet, a randomized trial should clarify the current data.

## Abbreviations

5-ALA: Five-aminolevulinic acid
AP: Anterior-posterior
BOLD: Blood oxygenation level dependent
CI: Confidence interval
CSF: Cerebrospinal fluid
CST: Corticospinal tract
CT: Computed tomography
DCS: Direct cortical stimulation
DTI: Diffusion tensor imaging
DWI: Diffusion-weighted imaging
EOR: Extent of resection
fMRI: Functional magnetic resonance imaging
GTR: Gross total resection
HGG: High-grade gliomas
IOM: Intraoperative neuromonitoring
KPS: Karnofsky performance status scale
MEG: Magnetoencephalography
MEP: Motor evoked potential
BMRC: British Medical Research Council
MRI: Magnetic resonance imaging
nTMS: Navigated transcranial magnetic stimulation
OR: Odds ratio
PET: Positron emission tomography
rMT: Resting motor threshold
SD: Standard deviation
STR: Subtotal resection
TMS: Transcranial magnetic stimulation
